# *RHO* Variants and Autosomal Dominant Retinitis Pigmentosa: Insights from the Italian Genetic Landscape

**DOI:** 10.3390/genes15091158

**Published:** 2024-09-02

**Authors:** Giulia Trastulli, Domenica Megalizzi, Giulia Calvino, Sarah Andreucci, Stefania Zampatti, Claudia Strafella, Carlo Caltagirone, Emiliano Giardina, Raffaella Cascella

**Affiliations:** 1 Genomic Medicine Laboratory UILDM, IRCCS Santa Lucia Foundation, 00179 Rome, Italy; 2 Department of Systems Medicine, Tor Vergata University, 00133 Rome, Italy; 3 Department of Biomedicine and Prevention, Tor Vergata University, 00133 Rome, Italy; 4 Department of Science, Roma Tre University, 00146 Rome, Italy; 5 Department of Clinical and Behavioral Neurology, IRCCS Fondazione Santa Lucia, 00179 Rome, Italy; 6 Department of Chemical-Toxicological and Pharmacological Evaluation of Drugs, Catholic University Our Lady of Good Counsel, 1010 Tirana, Albania

**Keywords:** retinitis pigmentosa, *RHO*, rare disease, NGS sequencing

## Abstract

Autosomal dominant retinitis pigmentosa (AD-RP) is caused by several genes, among which *RHO* is one of the most investigated. This article will be focused on *RHO* and its role in explaining AD-RP cases in the Italian population, taking advantage of the experience of the Genomic Medicine Laboratory UILDM at the Santa Lucia Foundation IRCCS. The retrospective evaluation of the distribution of *RHO* variants in the Italian patients with a clinical suspicion of RP pointed out eight variants. Of them, four variants (c.632A>T, c.1040C>T, c.1030C>T, c.383_392del) were pathogenic and made it possible to confirm the diagnosis of AD-RP in nine affected patients, highlighting a lower frequency (17%) of *RHO* variants compared to previous studies (30–40%). In addition, this study identified four variants classified as Variants of Uncertain Significance (VUS). In conclusion, the experience of the Genomic Medicine Laboratory provides an overview of the distribution of *RHO* variants in the Italian population, highlighting a slightly lower frequency of these variants in our cases series compared to previous reports. However, further studies on *RHO* variants are essential to characterize peculiar RP phenotypes and extend the spectrum of disease associated with this gene.

## 1. Introduction

Retinitis pigmentosa (RP, OMIM #268000) refers to a group of inherited dystrophies affecting the posterior segment of the eye [1]. The prevalence of RP ranges from 1 in 7000 to 1 in 3000 individuals, depending on the geographical area [2]. RP can be distinguished in non-syndromic forms occurring on their own without other clinical findings and syndromic ones, displaying ocular symptoms together with other neurosensory signs and systemic features [3]. RP is characterized by progressive photoreceptor cell death and retinal pigment epithelium (RPE) atrophy [4]. Phenotypic manifestations include nyctalopia, progressive vision loss leading to blindness (particularly at night), impairment of peripheral vision (rod photoreceptor dysfunction), development of tunnel vision, progressive reduction in the central visual field (cone dysfunction), and dyschromatopsia [2]. Generally, early-onset subtypes of RP progress more rapidly, with the condition often beginning around age 10. Visual impairment becomes evident and severe by age 40 to 50 [5].

In particular, non-syndromic RP displays a high level of heterogeneity in terms of genetic and inheritance patterns, variable penetrance, and expressivity [6]. RP is mostly a monogenic disease, and genetic variants lead to different biochemical changes in the retina. More than 100 genes have been linked to RP, which will likely increase over the years due to ongoing improvements in diagnostic testing techniques (RetNet, https://sph.uth.edu/RetNet/; accessed on 22 July 2024).

RP-associated genes encode proteins that play crucial roles in phototransduction, the visual transduction cascade, and photoreceptor transcription and structure [7]. Despite the genetic heterogeneity, most RP cases exhibit Mendelian inheritance patterns. RP is primarily categorized into three groups based on the mode of transmission, including Autosomal Dominant RP (AD-RP, 15–25%), Autosomal Recessive RP (AR-RP, 5–20%), and X-linked RP (XL-RP, 10–15%). Although rare, RP can also be inherited by mitochondrial or digenic patterns [8,9,10].

AD-RP is generally the least severe form of RP, with the onset of symptoms in adulthood and the preservation of visual acuity [7]. Several causative genes have been associated with AD-RP, including *RHO*, *PRPF31*, *PRPH2*, *RP1*, *IMPDH1*, *PRPF8*, *NR2E3*, *snRNP200*, *KLHL7*, *CRX*, *PRPF3*, *TOPORS*, *ADIPOR1*, *ARL3*, *CA4*, *FSCN2*, *GUCA1B*, *HK1*, *IMPG1*, *KIF3B*, *NRL*, *PRPF4*, *PRPF6*, *RDH12*, *ROM1*, *RP9*, *SEMA4A*, *SPP2*, and many others [6].

AR-RP is often linked to an earlier onset of symptoms and a more severe phenotype compared to AD-RP. While over 50 genes are known to cause AR-RP, only a few (such as *ABCA4*, *RPE65*, *PD56* complex, *PDE6A*, *PDE6B*, *CNGA1*, *RP25*, *CRB1*, *CERKL*, and *SAG*) account for the majority of common variants. The remaining genes are rare, each responsible for ≤1% of cases [6].

XL-RP is the most severe form of the disease, with the earliest onset and most rapid progression. In XL-recessive forms, affected males typically develop visual symptoms early in life, often experiencing night blindness before age 20 and progressing to significant visual impairment by their fourth decade [7]. In XL-dominant forms, affected females usually have a milder phenotype [11]. Two main genes, *RPGR* and *RP2*, are implicated in most XL-RP cases, accounting for 85–95% of all XL-RP forms.

The extensive heterogeneity of RP can complicate the clinical characterization of patients and, thereby, the management and treatment [3]. The differential diagnosis between RP and other inherited retinal dystrophies can be challenging due to overlapping symptoms. In such cases, genetic counseling and molecular testing proved to be fundamental for providing an accurate diagnosis and prognosis and accessing clinical trials in selected cases [12].

One of the most extensively studied genes involved in AD-RP development is *RHO* (OMIM *180380, 3q22.1) [13,14,15,16]. The *RHO* gene consists of five exons and encodes the rhodopsin protein (348 amino acids), characterized by seven transmembrane domains. This protein is synthesized in the inner segments of rod cells and subsequently transported to the outer segments [17]. Rhodopsin is a photopigment found in retinal rods, essential for vision in low-light conditions. Upon exposure to light, this transmembrane protein undergoes a conformational change that triggers the visual signal transduction cascade. Rhodopsin is crucial for visual processing, and minor disruptions in gene transcription, translation, protein folding, or transport can impair vision.

Previous in-depth characterization of *RHO* variants revealed distinct consequences for protein structure and function [17,18]. Approximately 30–40% of AD-RP cases can be explained by pathogenic variants of *RHO* [19]. It can also be responsible for dominant congenital stationary night blindness (CSNB) [20] and AR-RP [19,21,22], although these are rarer conditions compared to AD-RP [22,23].

A total of 439 variants associated with RP and related phenotypes have been described as Pathogenic, Likely Pathogenic, and Variant of Uncertain Significance (VUS). (https://www.ncbi.nlm.nih.gov/clinvar/?term=RHO%5Bgene%5D&redir=gene, last accessed on 22 July 2024, summarized in Table S1).

In particular, six variants have been documented in AR-RP cases [24]. Among them, four variants were missense (c.448G>A, p.E150K; c.931A>G, p.K311E; c.1031A>G, p.Q344R and c.759G>T, p.M253I) [21] and two were nonsense (c.482G>A, p.W161X; c.745G>T, p.E249X) [22,25]. These variants are all pathogenic, as described in the Franklin database (https://franklin.genoox.com/clinical-db/home, accessed on 22 July 2024), except for c.931A>G, which is classified as a VUS. This is very rare and it has been described in a sporadic patient with RP in the Chinese population [26]. The c.448G>A mutation is most prevalent in the South Asian population, with a percentage of 0.0003920 described in gnomAD. Conversely, the variants c.1031A>G (p.Q344R), c.759G>T (p.M253I), c.482G>A (p.W161X), and c.745G>T (p.E249X) are primarily described in the European population with allele frequencies below 0.000071.

Concerning AD-RP, the c.1040C>T variant is the most recurrent causative variant observed in patients from worldwide populations [27].

Given these premises, this article will focus on the contribution of *RHO* variants in explaining AD-RP cases in the Italian population, taking advantage of the long-lasting experience of the Genomic Medicine Laboratory UILDM at the Santa Lucia Foundation IRCCS in diagnosing such disorders.

## 2. Materials and Methods

### 2.1. Cohort Description

This study was conducted on a total of 752 patients who underwent genetic testing for inherited retinal dystrophies, including macular dystrophy, cone dystrophy, cone-rod dystrophy, and RP at the Genomic Medicine Laboratory UILDM of the Santa Lucia Foundation IRCCS, between 2016 and 2023. A retrospective revision of 752 patients sent to the laboratory for diagnostic purposes pointed out 261 cases accessing the laboratory with a clinical suspicion of RP. These patients displayed a Female:Male ratio of 47:39 and an average age of 44.7 ± 16.83 years.

### 2.2. DNA Purification and Quantification

The genomic DNA of patients was extracted from buccal swabs. Genomic DNA extraction was carried out utilizing the MagPurix^®^ 12A Instrument (Resnova, Rome, Italy) with the MagPurix^®^ Forensic DNA Extraction Kit (Resnova, Rome, Italy) for buccal swab samples.

A DS-11 FX spectrophotometer (DeNovix, Resnova) was employed to assess the quality and concentration of each DNA sample. Concentration ranges of 10–150 ng/μL and A260/230 and A260/280 ratios included between 1.7 and 1.9 were considered as good parameters for DNA samples.

### 2.3. Whole Exome Sequencing (WES) and Variant Analysis

Patients’ DNA samples were analyzed using Whole Exome Sequencing (WES). Concerning WES analysis, the Illumina^®^ Next-Seq 550 system (Illumina, San Diego, CA, USA) was utilized. In particular, 30–50 ng/μL of DNA was employed for library preparation by means of Illumina^®^ DNA Prep with the Enrichment and Tagmentation kit according to the manufacturer’s instructions. The obtained libraries were sequenced at 2 × 100 bp and the sequencing quality of the resulting data was expected to reach a quality score >30 (Q30) for ∼80% of total called bases. The genetic test was performed using WES analysis, followed by a study of 87 genes responsible for inherited retinal dystrophies (listed in Table S2). The resulting variants were visualized by Integrated Genome Viewer (v.2.7.2) and functionally annotated by means of BaseSpace Variant Interpreter (v. 2.15.0.110, Illumina, Illumina, San Diego, CA, USA), using GRCh37 as genome build. Annotated variants were prioritized considering the type (nonsense, missense, frameshift, splicing, loss or gain of function), frequency in publicly available reference database (GnomAD), localization in regulatory regions, and their pathogenicity scores through bioinformatic predictive tools. The effect of the variants on protein function and structure was evaluated with the Varsite prediction tool (https://www.ebi.ac.uk/thornton-srv/databases/VarSite, accessed on 22 July 2024). The variants were classified according to the ACMG Standards and Guidelines.

## 3. Results

**Figure 1:**
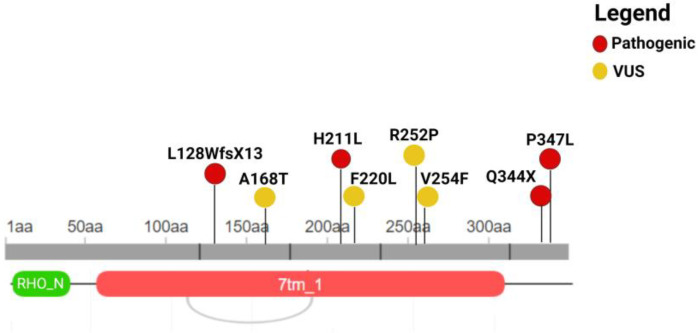
Illustration of the localization of the variants at the protein sequence level. Coding variants are reported with their protein nomenclature. The figure was built by retrieving the domain localization and visualization from Uniprot and Decipher, respectively. RHO_N = Rhodopsin_N; 7tm_1 = 7 transmembrane receptor (rhodopsin family). This figure was created with Biorender.com (Toronto, Canada).

The WES analysis gave special attention to the variants identified in the *RHO* gene. As a result, eight single-nucleotide variants (SNVs) were detected in 14 subjects, whereas the remaining individuals were of wild type for variants of *RHO* or for benign variants. Among them, four variants (c.632A>T, p.H211L; c.1040C>T, p.P347L; c.1030C>T, p.Q344X; c.383_392delTGGTGGTCCT, p.L128WfsX13) were classified as pathogenic and four variants (c.755G>C, p.R252P; c.760G>T, p.V254F; c.502G>A, p.A168T and c.660T>G, p.F220L) as VUS according to ACMG guidelines [28,29]. The identified variants are summarized in Table 1 and Figure 1.

As expected, the c.1040C>T was the most prevalent causative variant identified in six patients of our cases. Among these patients, one was a sporadic case and two were familial cases with multiple affected members. The sporadic case was a patient with the VUS c.502G>A. In one family with a father and two daughters, the c.1040C>T variant was present in all three affected individuals. The other familial case included the proband exhibiting symptoms of nyctalopia at the age of five and their parents. The variant was found in the affected proband and his father in this family. Altogether, the identification of the c.1040C>T at heterozygous state confirmed the suspicion of AD-RP in all these cases.

The c.1030C>T variant is described in LOVD (https://www.lovd.nl/, accessed on 22 July 2024) and ClinVar (https://www.ncbi.nlm.nih.gov/clinvar/, accessed on 22 July 2024) as pathogenic and was found at heterozygous state in one sporadic patient, consistently with the clinical suspicion of AD-RP.

In line with the clinical suspicion of AD-RP, the c.632A>T and c.383_392del variants were identified in two single sporadic patients at heterozygous state. Their allele frequencies are very low and absent in the gnomAD database. The c.632A>T is a missense variant that substitutes a histidine with a leucine at codon 211 of the protein. This amino acid change is highly unfavored in terms of conserved amino acid properties, and it is predicted to have a substantial impact on protein function. The c.383_392del is a deletion that results in a shift of reading frame and, consequently, the introduction of a premature stop codon.

Concerning the variants classified as VUS, the c.755G>C is a missense variant described in ClinVar and LOVD. The frequency of the variant is 0.0001859 in the European population and 0.00008841 in the general population. In our case series, this variant was heterozygous in three single patients with a clinical suspicion of AD-RP. The analysis of this variant by the VarSite application showed that the change from an arginine to a proline side chain at codon 252 of the protein is a large one and is highly unfavored in terms of conserved amino acid properties. Therefore, this amino acid change may significantly impact the protein function and structure.

Another missense variant classified as VUS is the c.502G>A that was identified with the c.1040C>T in a single sporadic patient. The variant consists of the substitution of threonine with alanine at codon 168 and VarSite does not predict a significant effect on protein function. The c.502G>A has an allele frequency of 0.00003100 in the European population and 0.00001415 in the general population.

In addition, the c.660T>G variant was identified in a single case with a clinical suspect of AD-RP and was described as VUS in ClinVar but as Likely Pathogenic in LOVD. The variant displays a frequency of 0.00003522 in the European population and 0.00004378 in the general population. The missense variant consists of the substitution of phenylalanine with a leucine, and a consultation of VarSite predicted that such a change is a minor one and would not be expected to affect the protein’s function.

The c.760G>T variant was detected in one case with a clinical suspicion of AD-RP, and it was not reported in frequency or genetic databases. This is a missense variant leading to a substitution of valine to phenylalanine. Following the VarSite consultation, this residue change is unfavored in conserved amino acid properties, so it may affect the protein’s function and structure.

## 4. Discussion

RP is a heterogeneous group of inherited retinal dystrophies characterized by progressive photoreceptor degeneration, leading to vision loss [1]. Causative variants in more than 100 genes have been reported in RP [24], out of which the *RHO* gene is one of the most investigated. Rhodopsin is vital in vision; one small mistake in gene transcription, translation, folding processing, or delivery to designated places may lead to vision damage [19]. Among 439 *RHO* variants described as Pathogenic, Likely Pathogenic, and VUS [24], 340 are missense and nonsense mutations associated with RP and related phenotypes [19].

In our cohort, the c.1040C>T variant was identified in six patients and consists of substituting a proline with a leucine at codon 347, a critical site in the rhodopsin protein essential for the visual transduction process [30]. It has been identified in several ethnic groups, including Japanese [31], Lithuanian [32], Chinese [33], and Spanish [34] populations. Research studies have shown that patients with the c.1040C>T variant usually display an early onset of disease with night blindness, followed by severe and progressive degeneration of rods and cones, culminating in significant vision loss by the fifth decade of life [27]. The c.1040C>T variant is often characterized by an AD phenotype, as described by Massof and Finkelstein (1993) [35], with early night blindness and widespread rod dysfunction, while central cone function may initially be preserved [27]. However, there is notable intra- and interfamilial variability in the severity and progression of vision loss among patients with this mutation [34].

The c.1030C>T variant was found at heterozygous state in one sporadic patient and has been extensively studied, revealing distinct pathogenesis and clinical manifestation characteristics. Generally, this variant is associated with mild phenotypes and slow progression of AD-RP [36]. The variant results from the conversion of codon 344 from glutamine to an early stop codon, truncating the protein and removing the trafficking signal VAPA, which leads to rhodopsin mislocalization and subsequent cellular apoptosis [37].

The c.632A>T and c.383_392del variants have been classified as pathogenic by ACMG criteria and have not been described in the literature and genomic databases, except for the c.632A>T reported in LOVD.

As expected, clinical variability and severity was observed considering the different pathogenic *RHO* variants. This is in line with the clinical severity and progression of AD-RP, which can vary significantly among patients. This variability can be influenced by several factors, including age, genetic modifiers, and external agents. For instance, the c.1040C>T variant is usually associated with a severe phenotype, often characterized by early onset and rapid progression of symptoms [34]. Conversely, the c.1030C>T variant has been associated with a milder phenotype, typically resulting in late-onset nyctalopia around the age of 20, followed by gradual loss of visual acuity and field [38].

Regarding the VUS identified in our case cohort, the c.755G>C variant was found in three single patients with a clinical suspicion of AD-RP. This variant has been described in two studies associated with inherited retinal dystrophy phenotypes [39], so it may play a role in the pathogenesis of AD-RP. Concerning the c.502G>A variant, its classification is conflicting since it is described as VUS in ClinVar and likely benign in LOVD. This variant was identified with the c.1040C>T in a single sporadic patient. Interestingly, it has been described in association with AD-RP in a study conducted on a group of Korean patients [40]. Given the co-occurrence of the c.502G>A and the c.1040C>T variants in a single sporadic patient, further investigation may be helpful for a better elucidation of the role of the c.502G>A variant as a contributing factor in RP.

The c.660T>G and c.760G>T variants were found in two single patients with a clinical suspicion of disease. The c.660T>G was reported in genetic databases such as ClinVar and LOVD, supporting its possible involvement in AD-RP, although it is not reported in literature studies. Concerning the c.760G>T variant, it is not found in frequency and genetic databases, although it was described in a study conducted by Mark P. Krebs et al. [41]. In 2010, the authors conducted a study exploring the molecular basis of rhodopsin-associated RP in the context of pharmacological rescue with 11-cis retinal. They hypothesized that rhodopsin-associated RP is a protein misfolding disease (also known as a conformational protein disease), in which the misfolding or misassembly of a mutant protein alters its cellular fate and induces cell death [42,43]. Specifically, they observed that the c.760G>T variant was the least misfolded and did not co-segregate with the disease phenotype. Given these results, the authors concluded that this variant was non-pathogenic. However, other pathogenic mechanisms and, subsequently, their contributing roles in AD-RP cannot be excluded for this variant. In summary, while VUS in the *RHO* gene may potentially alter protein structure and function, their exact role in AD-RP pathogenesis remains uncertain. Further functional studies and clinical correlations are necessary to clarify the impact of these variants on disease mechanisms and to determine their significance in the context of AD-RP.

Literature studies reported a 30–40% frequency of causative variants in *RHO* [24] in the United States (US) and the United Kingdom (UK) populations, in contrast to the lower percentages reported among European populations, including 20% described in Spain [44], 16% in Germany [45], 16–26% in Italy [46], and 10% in Southern France [47]. In this study, the experience of the Laboratory of Genomic Medicine UILDM at Fondazione Santa Lucia IRCCS in the diagnosis of inherited retinal dystrophies was exploited to evaluate the distribution of *RHO* variants in the Italian patients. In particular, the whole cohort (n = 752) available at the laboratory was initially filtered for patients with a clinical suspicion of RP, which resulted to be 261. Of them, 53 patients (20%) had a clinical suspicion of AD-RP, in line with literature studies reporting similar data concerning the occurrence of this RP form [46,48]. Among these patients, 9 out 53 received a diagnosis of AD-RP due to a causative variant of *RHO*, revealing thereby a 17% frequency of *RHO* variants in our Italian cohort. This result was similar to the frequency previously reported among European groups, supporting the lower frequency of *RHO* variants in these populations compared to US and UK ones.

In 2005, Ziviello et al. [46] conducted a study on 43 Italian families to investigate the presence of variants in 12 genes (*RHO*, *RDS*, *RP1*, *IMPDH1*, *PRPF31*, *CRX*, *NRL*, *FSCN2*, *HPRP3*, *RP9*, *CA4*, and *PRPF8*) associated with AD-RP. This study focused on these 12 genes as they were the only known disease-causing genes at that time. Variants of the *RHO* gene were found in seven families (16% of cases), thereby representing the most prevalent gene involved in the pathogenesis of AD-RP. Interestingly, this prevalence was lower than the 25–50% reported in the US and UK [49], although it was more comparable to that observed in other European populations, such as Spain, southern France [34,50], and in the Far East [51,52,53]. Among the variants observed by Ziviello and coworkers, the c.403C>T (R135W) missense variant was the most frequently detected (reported in four families), followed by the c.1040C>T variant (found in two families). Interestingly, the c.1040C>T variant was the most frequently observed in our cohort as well (6/53). Additionally, it appears to be widely reported in Japanese [31], Lithuanian [32], Chinese [53], and Spanish [34] populations, thereby representing the most frequent *RHO* variant responsible for AD-RP [34]. In more recent research studies [12,54], it still appears to be the most frequent variant associated with AD-RP in the Italian population. A similar phenotype has been described for this variant among different populations [52], including early onset and rapid progression of disease [51].

In 2010, Audo et al. [50] studied the prevalence of *RHO* variants in French patients with AD-RP and cone dystrophy. As a result, they found a 16.5% frequency of such variations, which was consistent with reports from other European cohorts [47]. In 2015, another study [34] focused on the Spanish population, revealing a 21% frequency of *RHO* variants as responsible for AD-RP cases, in line with previous data (19.5%) observed in this country [44]. The results of these two studies are consistent with our findings, even though they primarily employed Sanger sequencing or genotyping microarrays.

Pierrottet et al., in 2014 [49], published a review of the mutations related to syndromic and non-syndromic RP in Italy, among which 22 different heterozygous mutations were identified in 33.9% of patients. In this study, 22.2% of cases had a confirmed diagnosis of AD-RP, identifying two *RHO* variants (11%), namely the c.545G>T (p.G182V) and c.1040C>T variants. The c.545G>T variant is a missense mutation in exon 3 of *RHO*. This variant is described in ClinVar as pathogenic and, in the Italian [49], Chinese [53] and Spanish [34] populations. In the Chinese population, this variant has been associated with an onset of blindness in early childhood [53]. In our cohort, this variant was not identified. The c.1040C>T variant has been previously discussed.

In 2021, Colombo et al. [12] described the molecular epidemiology of non-syndromic RP and Usher Syndrome in Italian patients. Among 591 probands, the results showed a prevalence of 10% (59 probands) for AD-RP. The most frequent disease-causing genes in AD-RP probands were *RHO* (29%), which differ from the frequencies found in this study, as well as in those by Pierrotet et al. 2014 and Ziviello et al. [46]. In 2022, Karali et al. [54] conducted a retrospective epidemiological study to determine the genetic basis of inherited retinal diseases in a large Italian cohort (n = 2790). Over the years, various genotyping methods were employed, following the technical evolution of sequencing methodologies. Earlier analyses relied on single-gene tests (e.g., PCR on genomic DNA followed by Sanger sequencing) whenever the clinical phenotype and inheritance pattern strongly indicated a candidate gene. Since 2013, samples have been screened using high-throughput targeted sequencing. More recently, patient samples have been analyzed using customized panels of known retinopathy genes or WES. The authors reviewed the clinical records and genetic data of the selected patients and identified 2036 subjects (from 1683 families) with a potentially conclusive diagnosis. They identified a total of 1319 causal sequence variations in 132 genes, including 353 novel variants and 866 genotypes potentially amenable to therapeutic approaches. Of the total cases, 13.9% (n = 283) represented AD forms and included 72 patients with a causative *RHO* variant. In this study, the frequency of *RHO* variants explaining AD-RP cases was found to be of 25%.

Overall, this study provides an overview of the distribution of *RHO* variants in RP in the Italian population, highlighting a slightly lower frequency of causative variants compared to previous works performed in Italian and worldwide populations. In particular, this study identified a *RHO* causative variant in 17% affected patients with respect to the 30–40% estimated frequency reported in US and UK studies [12,55]. Nevertheless, the frequency observed in the present study was similar to that observed in other studies performed in European populations [34,46,50]. These findings further confirm *RHO* as one of the genes responsible for AD-RP, although it seems to be less frequent in the Italian population. Furthermore, this study emphasizes the importance of simultaneously testing for variants of *RHO* and other genes associated with AD-RP, to reduce the time for diagnostic response and improve its accuracy. Moreover, further studies on *RHO* variants may still be necessary to better characterize peculiar RP phenotypes and expand the knowledge of the disease spectrum associated with *RHO* variants. Identifying *RHO* gene variants holds significant promise for early diagnosis and personalized treatment strategies in AD-RP in the future. In this regard, genetic screening in families with a history of AD-RP can facilitate early diagnosis, allowing for timely interventions that may slow disease progression and improve patient outcomes [56]. Understanding the specific effect of genetic variants associated with AD-RP enables a more precise prediction of disease progression. For example, patients with causative variants of the *RHO* gene often present with a classic RP phenotype. Early detection of such alterations can guide the implementation of potential gene therapies and other targeted treatments [57]. On this subject, pharmacological chaperones and gene therapy have shown promise in addressing the function of mutant RHO proteins associated with AD-RP. Pharmacological chaperones like SRD005825, YC-001, and TUDCA have demonstrated the ability to stabilize mutant RHO proteins, enhancing their function and improving photoreceptor survival in preclinical models. These compounds work by improving protein folding and protecting against retinal degeneration. Despite these promising findings, additional clinical trials are required to validate their efficacy and safety in human subjects [58,59,60]. Gene therapy approaches, including gene augmentation and CRISPR/Cas9-based strategies, aim to correct or compensate for *RHO* detrimental variants. While there have been successes in introducing wild-type genes to counteract the effects of pathogenic variants, the results have been variable and often limited. CRISPR/Cas9 holds potential use for more versatile and durable treatments, but it is still under investigation [61]. In summary, while targeted therapies such as pharmacological chaperones and gene therapy offer significant hope for rescuing the function of mutant RHO proteins, further research and clinical validation are essential to establish their effectiveness and optimize treatment strategies for RP [19]. Furthermore, greater attention should be given to genes involved in AD-RP other than *RHO* and to their possible interactions in the disease pathogenesis. While *RHO* is a principal gene implicated in AD-RP, other genes (such as *PRPF31*, *PRPH2*, and *RP1*) also play significant roles in the disease. *PRPF31*, for instance, represents one of the most common genetic cause of AD-RP in many populations [62]. Causative variants of *PRPF31* have been shown to hamper the pre-mRNA splicing of the *RHO* gene, suggesting the abnormal splicing of transcripts involved in the retinal function as the pathogenic mechanism underlying AD-RP [63,64]. PRPH2 is a retina-specific transmembrane protein that is crucial for the morphogenesis and maintenance of photoreceptors (rod and cone) disc outer segments. Given its role as structural protein, disease-causative variants of *PRPH2* have been described in multiple phenotypes, including AD-RP. Interestingly, *RHO* and *PRPH2* have been shown to interact together with *CNGB1*, forming a complex that is essential for rod outer-segment structural stability [65]. In addition, a recent work described the reduction in RHO levels as a therapeutic strategy for *PRPH2* disease-causing variants [66]. *RP1* is another gene known to cause AD-RP and it is a photoreceptor-specific microtubule protein. Detrimental variants of *RP1* have been shown to cause outer-segment disorganization, photoreceptor dysfunction, and degeneration [63,64]. Interestingly, the RP1 protein has been suggested to play a role in regulating the rhodopsin transport between inner and outer segments of photoreceptors [63]. Overall, these findings further emphasize the need for further research to elucidate potential interactions between *RHO* and other genes as well as their implications not only as disease-causative factors but also as possible disease modifiers and therapeutic targets.

## Figures and Tables

**Table 1:** Description of the identified *RHO* variants (NM_000539.3). Na: not available; ACMG Classification: American College of Medical Genetics and Genomics Classification; VUS: Variant of Uncertain Significance.

Genomic Position	Exon	Nucleotide Coding	Protein Coding	Rs	Effect	ACMG Classification
3:129251195	3	c.632A>T	p.H211L	Na	missense	Pathogenic
3:129252554	5	c.1040C>T	p.P347L	rs29001566	missense	Pathogenic
3:129252544	5	c.1030C>T	p.Q344X	rs104893778	nonsense	Pathogenic
3:129249740_129249749	2	c.383_392del	p.L128WfsX13	Na	frameshift	Pathogenic
3:129251439	4	c.760G>T	p.V254F	Na	missense	VUS
3:129249859	2	c.502G>A	p.A168T	rs574202023	missense	VUS
3:129251223	3	c.660T>G	p.F220L	rs141956356	missense	VUS
3:129251434	4	c.755G>C	p.R252P	rs765438313	missense	VUS

## Data Availability

All data presented in this study are available in the main text and in its supplementary file. Any other requests of data should be directed to the corresponding author.

## Supplementary Materials

The following supporting information can be downloaded at: https://www.mdpi.com/article/10.3390/genes15091158/s1, Table S1: List of the 439 variants of the *RHO* gene with their characteristics described in ClinVar; Table S2: List of the genes associated with inherited retinal dystrophies.
